# Cholesterol, Oxysterol, Triglyceride, and Coenzyme Q Homeostasis in ALS. Evidence against the Hypothesis That Elevated 27-Hydroxycholesterol Is a Pathogenic Factor

**DOI:** 10.1371/journal.pone.0113619

**Published:** 2014-11-21

**Authors:** Anna Wuolikainen, Jure Acimovic, Anita Lövgren-Sandblom, Paolo Parini, Peter M. Andersen, Ingemar Björkhem

**Affiliations:** 1 Computational Lifescience Cluster (CLiC), Umeå University, Umeå, Sweden; 2 Department of Chemistry, Umeå University, Umeå, Sweden; 3 Department of Laboratory Medicine, Karolinska Institute, Huddinge, Sweden; 4 Department of Pharmacology and Clinical Neuroscience, Umeå University, Umeå, Sweden; National Institute of Health, United States of America

## Abstract

High plasma levels of cholesterol have been suggested to be neuroprotective for the degenerative disease amyotrophic lateral sclerosis (ALS) and to be associated with increased survival time. The gene encoding cholesterol 27-hydroxylase, *CYP27A1*, was recently identified as a susceptibility gene for sporadic ALS. A product of this enzyme is 27-hydroxycholesterol. We investigated plasma samples from 52 ALS patients and 40 control subjects (spouses) regarding cholesterol homeostasis, lipid profiles, and coenzyme Q. Eleven of the patients carried mutations in *C9orf72* and seven in *SOD1*. Plasma levels of 27-hydroxycholesterol were significantly lower in male patients with ALS than in controls. It was not possible to link the reduced levels to any specific mutation, and there was no significant correlation between 27-hydroxycholesterol and survival. With normalization for diet using the spouses, a correlation was found between survival and total cholesterol, very low density lipoprotein cholesterol, low density lipoprotein cholesterol, and coenzyme Q. We conclude that cholesterol, 24S-hydroxycholesterol, 25-hydroxycholesterol, 27-hydroxycholesterol and lipid profiles in plasma are of limited prognostic value in individual ALS patients.

## Introduction

Amyotrophic lateral sclerosis (ALS) is a heterogeneous adult-onset neurodegenerative syndrome hallmarked by focal onset of wasting of skeletal muscles that becomes generalized within a few years. The patient eventually dies when the respiratory muscles become affected. Mutations in 27 genes have been associated with causing ALS [1] but the etiology is unknown in most of the patients. ALS has however also been reported to be associated with significant metabolic changes including hypermetabolism [2]–[3] and hyperlipidemia [4]. Noticeably, ALS patients with high BMI and/or diabetes mellitus appear in general to have a better prognosis [5]. According to studies of some authors [4]–[6] but not all [7], hyperlipidemia is correlated with longer survival in patients with ALS. In accordance with the contention that hyperlipidemia has a preventive effect, increasing the lipid content of the diet offers neuroprotection and extends survival in animal models of ALS [8]–[9]. In contrast, caloric restriction exacerbates the motor symptoms [10]. A number of studies have suggested that lowering the levels of plasma cholesterol by medication with statins may accelerate functional decline in ALS patients (for a review, see [11]). According to a recent meta-analysis including two case-control studies and one cohort study, however, the detrimental effects of statins are abolished when adjusted for age of onset and body mass index (BMI) [11]. It was concluded that existing data is still inconclusive to make scientifically supported conclusions with respect to the relation between ALS and statin use. Statins reduce cholesterol levels by inhibiting its synthesis. The possibility must be considered that cholesterol synthesis rather than plasma cholesterol level is related to ALS. To our knowledge this possibility has not been studied previously. Since cholesterol synthesis is balanced by degradation of cholesterol into bile acids, also the rate of conversion into bile acids may be of importance.

A possible link between statins and functional decline in ALS patients is coenzyme Q10. This compound is the electron acceptor for mitochondrial complexes I and II and is a powerful antioxidant. Q10 is formed in the mevalonate pathway and its level can be expected to be reduced by treatment with statin. In a study of 30 sporadic ALS cases (20 spinal, 10 bulbar) and 42 age- and sex-matched controls, the serum levels of coenzyme Q10 were normal in patients with ALS [12]. To our knowledge, this has not been confirmed by other groups.

Recently, a possible new link between cholesterol and cholesterol metabolism was reported. A large and comprehensive genome-wide association screening was performed in an attempt to find novel genetic variants and candidate genes for sporadic ALS [13]. Surprisingly the gene encodingsterol 27-hydroxlase, *CYP27A1*, was identified as a possible candidate gene for the disease. Cholesterol is a major substrate for this enzyme, and the product, 27-hydroxycholesterol, appears to accelerate neurodegeneration by several mechanisms (e.g., through diminished production of the “memory protein” Arc), as reviewed by Björkhem *et al.* [14]. Thus it was considered to be of interest to measure plasma levels of 27-hydroxycholesterol in ALS patients.

In the present work, we have further investigated the possibility that cholesterol homeostasis is abnormal in patients with ALS and that it is associated with prognosis. We have extended the study to include lipid profiling in different lipoprotein fractions as well as a marker for cholesterol synthesis (lathosterol) and a marker for metabolism of cholesterol into bile acids (7-alpha hydroxy-4-cholesten-3-one). Furthermore, we have measured plasma levels of 27-hydroxycholesterol. For reasons of comparison we also included measurement of two other oxysterols in plasma, 24S-hydroxycholesterol and 25-hydroxycholesterol. We further wanted to investigate if abnormal sterol metabolism was associated with genetic subtypes of ALS in particular with the most common ALS-causing genes Cu/Zn superoxide dismutase (*SOD1*) and the large GGGGCC-hexanucleotide repeat expansion in the *C9orf72* gene. In addition we measured circulating levels of coenzyme Q/Q10 in serum from the ALS patients and the spouses of the patients.

## Materials and Methods

### Patients

The study was performed in accordance with the Declaration of Helsinki (World Medical Association, 1964) and approved by the Ethical Review Board for Medical Research, Sweden (FEK UmU dnr 94–135; date of approval September 15, 1994). Having obtained written informed consent from the patients, we collected blood samples into EDTA-containing vacuum tubes and centrifuged them into plasma, buffy coat, and erythrocyte fractions. These were immediately frozen at −80°C until analysis. The blood was obtained from 52 ALS patients (23 females and 29 males) who had been diagnosed according to the EFNS consensus criteria [15], and 40 control subjects (19 females and 21 males), 35 of who were the spouses of some of the 52 patients studied. The other 5 control samples were from spouses of other ALS patients (who were not included in the study because they were taking medications or had other medical conditions that might interfere with interpretation of our analyses). Every attempt was made to match the patient and control populations for age and gender to get as uniform study cohorts as possible. This included also the storage time of the samples had been kept in the freezer. All participants had a standard oral diet at the time the blood samples were collected.

The patients were screened for a panel of ALS-causing genes. Eleven patients had large hexanucleotide-repeat expansion mutation in *C9orf72*, seven carried missense mutations in the gene encoding the free-radical scavenging enzyme *SOD1*, 1 had a mutation in the vesicle-associated membrane protein-associated protein B and C (*VAPB*), and two had mutations in the gene encoding fused in sarcoma (*FUS*). One individual with an A4V *SOD1* mutation was sampled 8 years prior to showing ALS symptoms and again after ALS had developed. Five patients were sampled longitudinally over time during the course of the ALS disease, one of which (a female patient) was followed regularly over 11 months (one occasion duplicated) to assess variability. Patient characteristics are summarized in Table S1 (found in File S1).

### Cholesterol, triglycerides, and lipoprotein profiles

Measurement of cholesterol and triglyceride content in the lipoprotein fraction (high density lipoprotein (HDL), low density lipoprotein (LDL), and very low density lipoprotein (VLDL) was performed with an automated gel filtration system (size exclusion chromatography) with online enzymatic measurements of cholesterol and triglycerides [16]. Total cholesterol and triglyceride values were calculated by addition of the content in the different fractions.

### Oxysterols

Oxysterols (24S-, 25-, and 27-hydroxycholesterol) were analyzed by isotope dilution mass spectrometry (MS) as described previously [17].

### Lathosterol

Lathosterol was analyzed by isotope dilution MS as described [18].

### 7α-Hydroxy-4-cholesten-3-one

7α-Hydroxy-4-cholesten-3-one was analyzed by liquid chromatography (LC)-MS-MS as described [19].

### Coenzyme Q

After oxidation of the reduced form of coenzyme Q10 as described [20], total coenzyme Q was quantitated by LC-MS as described previously [21].

All analytical data are summarized in table S2 (found in File S1).

### Statistical analysis

Comparisons between controls and ALS patients (females/males) were done for the measured parameters using a non-parametric test (Mann-Whitney U-test), calculated using an in-house script (R 2.15.2) and the first-collected sample from each subject (tables 1 and 2). A detailed overview of the parameters measured (including genetic subgroups) can be found online (Fig. S1–S5, File S1). Principal component analysis (PCA) [22] (SIMCA 13; Umetrics AB, Umeå, Sweden) was used to overview the measured variables. Significance of principal components was based on eigenvalue criteria (>2). Models for females and males were created separately due to gender differences in concentrations of the metabolites measured. Normalization was done for each individual by dividing all measured parameters by the patient's (or the control's) age or BMI. Females were normalized against age and males where normalized against BMI. Mann-Whitney U test p-values before and after age normalization in females (adjusting for differences between females in age, fold 0.9 ALS/controls) and BMI normalization in the males (adjusting for differences between males in BMI, fold 0.9 ALS/controls) can be found in table S3 (found in File S1). To correct for dietary factors, all ALS subjects who had their spouse included in the study were also normalized against the spouse. There were 21 pairs (22 pairs if two samples from the same patient were matched to the same sample from the spouse), of which 9 were female-to-male and 12 (or 13) were male-to-female. Orthogonal projections to latent structures-discriminant analysis (OPLS-DA) [23] was used to investigate what variables were able to differentiate males with ALS from male controls. The samples from the male subjects before and after diagnosis of ALS were compared for all parameters measured and the pre-diagnostic sample was predicted into the male OPLS-DA model (Fig. S6, File S1).

**Table 1:** Female subjects Average values ± standard deviation for parameters measured for female controls and ALS patients, fold change (ALS/controls), and p-values for females.

	Controls (n = 19)	ALS (n = 23)	Fold (ALS/Control)	p (Mann-Whitney)
	females	females	Females	Females
**Age** (years)	63.8±6.4	57.5±14.3	0.9	0.11
**BMI** (kg/m^2^)	23.3±3.1	23.4±4.2	1.0	0.82
**Total cholesterol** (mM)	6.0±1.2	6.3±1.5	1.0	0.37
**VLDL cholesterol** (mM)	0.8±0.3	0.8±0.5	1.0	0.94
**LDL cholesterol** (mM)	3.2±0.8	3.4±0.9	1.1	0.23
**HDL cholesterol** (mM)	2.1±0.8	2.1±0.6	1.0	0.69
**Total triglycerides** (mM)	1.1±0.4	1.1±0.4	1.0	0.70
**VLDL triglycerides** (mM)	0.6±0.4	0.6±0.4	1.0	0.95
**LDL triglycerides** (mM)	0.3±0.0	0.3±0.1	1.2	0.11
**HDL triglycerides** (mM)	0.2±0.1	0.2±0.1	1.1	0.25
**Lathosterol** (µg/mL)	1.0±0.6	1.2±0.7	1.2	0.23
**7-alfa-hydroxy-4-cholesten-3-one** (nmol/L)	37.6±27.8	48.8±40.7	1.3	0.43
**24S-hydroxycholesterol** (ng/mL)	61.9±14.1	71.2±16.7	1.1	0.12
**25-hydroxycholesterol** (ng/mL)	6.4±2.2	7.1±3.5	1.1	0.66
**27-hydroxycholesterol** (µg/mL)	0.14±0.03	0.14±0.04	1.0	0.77
**Coenzyme Q** (µg/mL)	1.5±0.3	1.5±0.3	1.0	0.63

**Table 2:** Male subjects Average values ± standard deviation for parameters measured for male controls, ALS patients, fold change (ALS/controls), and p-values for males.

	Controls (n = 21)	ALS (n = 29)	Fold (ALS/Controls)	p (Mann-Whitney)
	males	males	Males	Males
**Age** (years)	59.6±12.2	60.0±13.2	1.0	0.63
BMI (kg/m^2^)	27.4±2.7	24.2±3.3	0.9	0.40
**Total cholesterol** (mM)	5.6±1.0	5.7±1.2	1.0	0.79
**VLDL cholesterol** (mM)	1.0±0.4	0.8±0.4	0.8	**0.01**
**LDL cholesterol** (mM)	3.2±0.7	3.4±0.9	1.1	0.76
**HDL cholesterol** (mM)	1.4±0.3	1.6±0.4	1.1	0.18
**Total triglycerides** (mM)	1.4±0.4	1.1±0.5	0.8	**0.01**
**VLDL triglycerides** (mM)	0.9±0.3	0.6±0.4	0.7	**0.001**
**LDL triglycerides** (mM)	0.3±0.1	0.3±0.1	1.0	0.20
**HDL triglycerides** (mM)	0.2±0.1	0.2±0.1	1.0	0.22
**Lathosterol** (µg/mL)	1.5±0.8	1.1±0.5	0.8	0.18
**7-alfa-hydroxy-4-cholesten-3-one** (nmol/L)	68.3±92.9	45.9±45.6	0.7	0.30
**24S-hydroxycholesterol** (ng/mL)	61.3±12.6	63.4±15.1	1.0	0.67
**25-hydroxycholesterol** (ng/mL)	7.5±2.6	8.2±2.8	1.1	0.39
**27-hydroxycholesterol** (µg/mL)	0.2±0.05	0.16±0.05	0.8	**0.01**
**Coenzyme Q** (µg/mL)	1.8±0.3	1.6±0.41	0.9	0.07

Pearson's correlation coefficient, Spearman's correlation coefficient, and p-values for correlation were calculated using the statistical toolbox in MATLAB (R2013; Mathworks, Natick, MA) between the measured parameters and survival from the time point of sampling for all subjects with known survival status (a few patients were still alive). The factors were further normalized against body mass index (BMI, calculated as weight (kg)/height^2^ (m^2^)) and age, and significance testing and correlation to survival from the time point of sampling was re-calculated. Pearson correlations were further calculated between age and the measured parameters and between BMI and the measured parameters for female and males, controls and ALS. Significant correlations are presented in table S4, File S1. OPLS modeling [24] was used for prediction of survival from sampling of the diet-normalized ALS group, and the model was optimized for significance. Cross-validated analysis of variance was used to assess and optimize the significance of the OPLS model. Individual subjects who had samples collected over time were overviewed for variation in the measured parameters. A female patient who was followed over 11 months was overviewed in the measured parameters to overview variation over time.

## Results

### Comparison of measured parameters between ALS patients and controls

Because of known gender differences in the factors measured and the finding that the male ALS patients had lower BMI than the controls (fold ALS/controls: 0.9), and that the female ALS patients were generally younger (fold ALS/controls: 0.9), we present the data separately for the genders. Table 1 and 2 give the basal characteristics of the patients at the first time of sampling of the plasma. Tables for age (females) and BMI (males) adjusted Mann-Whitney U test p-values can be found in table S3 (found in File S1).

There was no significant difference between female patients and controls for any of the different parameters measured (table 1). When adjusted for age, the levels of LDL triglycerides (p = 0.01), HDL triglycerides (p = 0.01), lathosterol (p = 0.05), LDL cholesterol (p = 0.04) and 24S-hydroxycholesterol (p = 0.0004) were significantly increased in ALS patients compared to the controls.

**Figure 1:**
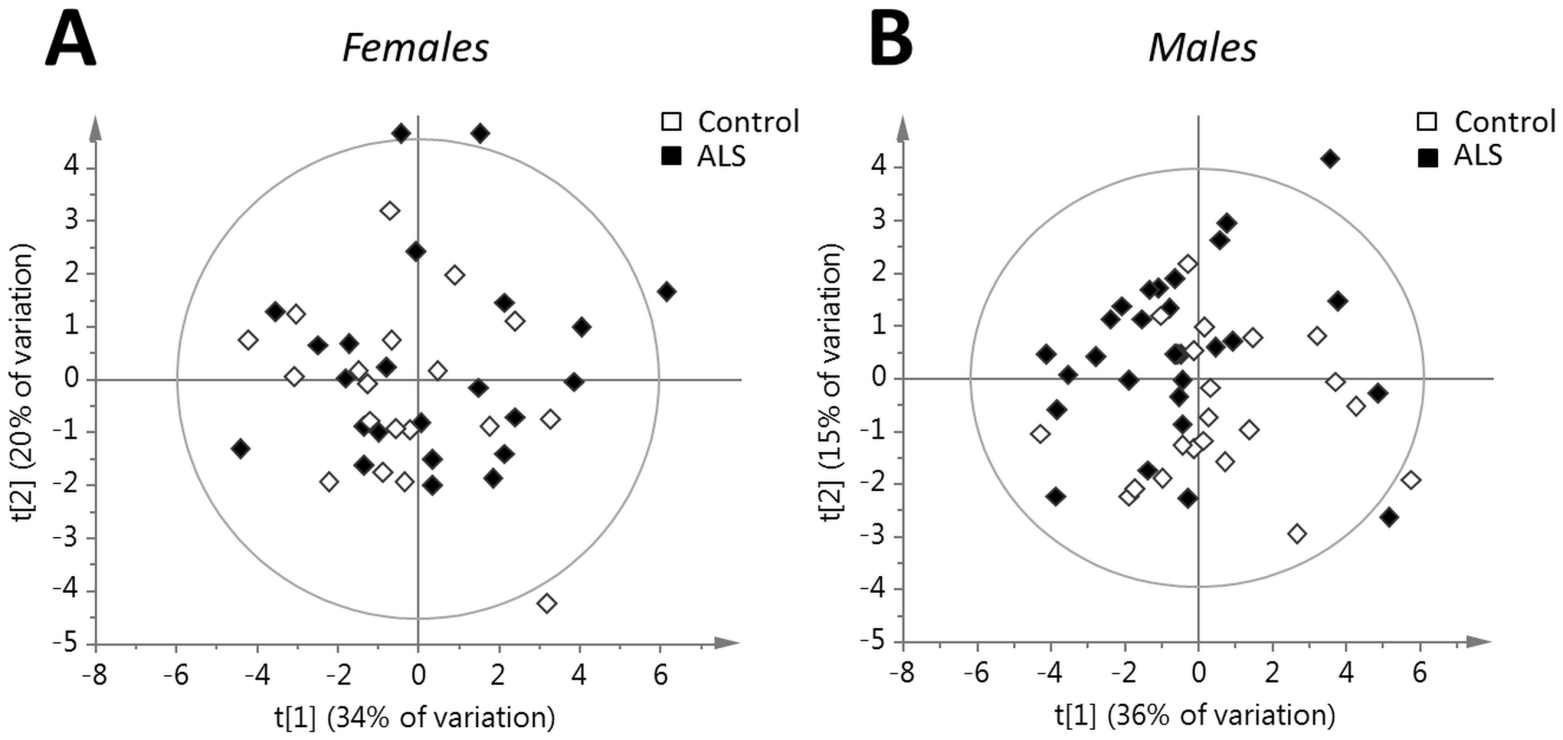
Multivariate analysis using PCA of the parameters in tables 1 and 2 (with BMI and age excluded) using two significant principal components based on eigenvalue criteria (>2). A. Females with ALS overlapped with female controls in the measured parameters. (t [1], 34% of variation vs. t [2], 20% of variation; R2 = 0.67; Q2 = .25). B) The male ALS group was shifted from the male controls in the measured parameters. (t [1], 36% of variation vs. t [2], 15% of variation; R2 = 0.51; Q2 = 0.22).

A number of significant differences were observed between male patients and controls (table 2). VLDL cholesterol (p = 0.01), VLDL triglycerides (p = 0.01), and total triglycerides (p = 0.01) were found to be lower in ALS patients than in the controls. A correlation between triglyceride levels and BMI would be expected. BMI was found to be non-significantly lower in ALS (p = 0.40). Correlations between the measured factors and age and BMI were investigated. Only 27-hydroxy cholesterol was found to correlate with BMI in controls and only in the female subjects. Various factors including lathosterol and coenzyme Q correlated to age and BMI in the ALS group. All the significant correlations (p<0.05) between the measured parameters and age and BMI can be found listed in table S4 (found in File S1). After normalization for differences in BMI, VLDL cholesterol (p = .003), total triglycerides (p = 0.05), and VLDL triglycerides (p = .004) were still found to be significantly different between ALS patients and controls. A notable finding was that the level of the oxysterol 27-hydroxycholesterol was significantly lower in the male patients (p = 0.008) than in the male controls (fold ALS/controls: 0.81). The lower levels could not be explained by a correlation with cholesterol. When testing 27-hydroxycholesterol in males normalized against BMI, the difference was still significant (p = .03). A PCA (Fig. 1) to overview variance of the parameters in tables 1 and 2 (age and BMI excluded) showed that about 50–60% of the structural variance in the parameters measured for both males (R2 = .51) and females (R2 = 0.67) could be explained. About 20% was useful for prediction (Q2 = 0.22 for the male model; Q2 = 0.25 for the female model). Females with ALS overlapped with female controls in the measured parameters while the male ALS group shifted from the male controls.

There were no obvious differences between patients with different clinical variants of ALS (spinal onset/bulbar onset and Vulpian-Bernhardts variant/others) or genetic variants (*C9orf72/SOD1*/others; shown in Fig. S1–S5, File S1).

Plasma from the male patient with an A4V *SOD1* mutation sampled 8 years before developing ALS showed decreased total cholesterol, VLDL cholesterol, VLDL triglycerides, and 27-hydroxycholesterol. When the measured parameters were used to predict which class the sample belonged to (control or ALS) using an OPLS-DA model based on males, the prediction ended up closer to the ALS group than to the control group (Fig. S6, File S1). Thus, the pre-morbid sample taken 8 years prior to diagnosis was more similar to samples from male ALS patients than to samples from male controls.

### Comparison between the parameters and time of ALS survival

Table 3 summarizes the correlation between time of survival and the levels of the measured parameters in the male patients, female patients, and combined ALS patients.

**Table 3:** Correlation to survival Pearson's correlation for the parameters plotted against survival from time of sampling for all subjects and Spearman's correlation for the parameters plotted against survival from time of sampling for females and males.

	Pearson's correlation vs. survival time from sampling (months)	p-value	Spearman's correlation vs. survival time from sampling (months)	p-value	Spearman's correlation vs. survival time from sampling (months)	p-value
	*all (N = 46)*		*females (N = 24)*		*males (N = 22)*	
**Age**	−0.2	0.25	0.1	0.79	−0.4	**0.04**
**BMI**	0.2	0.20	0.0	1.0	0.4	0.07
**Total cholesterol**	0.3	0.10	0.3	0.21	0.4	0.08
**VLDL cholesterol**	0.2	0.15	0.4	0.08	0.3	0.16
**LDL cholesterol**	0.2	0.29	0.0	1.0	0.5	**0.01**
**HDL cholesterol**	0.2	0.14	0.4	0.08	0.1	0.66
**Total triglycerides**	0.2	0.16	0.4	0.09	0.1	0.67
**VLDL triglycerides**	0.2	0.13	0.3	0.11	0.3	0.13
**LDL triglycerides**	−0.2	0.58	−0.1	0.54	−0.4	0.10
**HDL triglycerides**	0.4	**0.01**	0.7	**0.0001**	−0.1	0.63
**Lathosterol**	0.4	**0.01**	0.4	0.08	0.2	0.40
**7 alfa-hydroxy-4-cholesten-3-one**	0.0	1.0	0.0	1.0	−0.1	0.83
**24S-hydroxycholesterol**	0.3	0.07	0.1	0.72	0.2	0.34
**25-** **hydroxycholesterol**	0.0	1.0	0.0	1.0	−0.4	0.11
**27-** **hydroxycholesterol**	0.3	0.06	0.3	0.12	0.1	0.76
**Coenzyme Q**	0.2	0.13	0.1	0.52	0.4	**0.04**

The following parameters did not significantly correlate with time of survival in any of the two genders (p>0.05): BMI, total cholesterol, VLDL cholesterol, HDL cholesterol, VLDL triglycerides, LDL triglycerides, total triglycerides, 7α-hydroxy-4-cholesten-3-one, 24S-hydroxycholesterol, 25-hydroxycholesterol, or 27-hydroxycholesterol.

**Figure 2:**
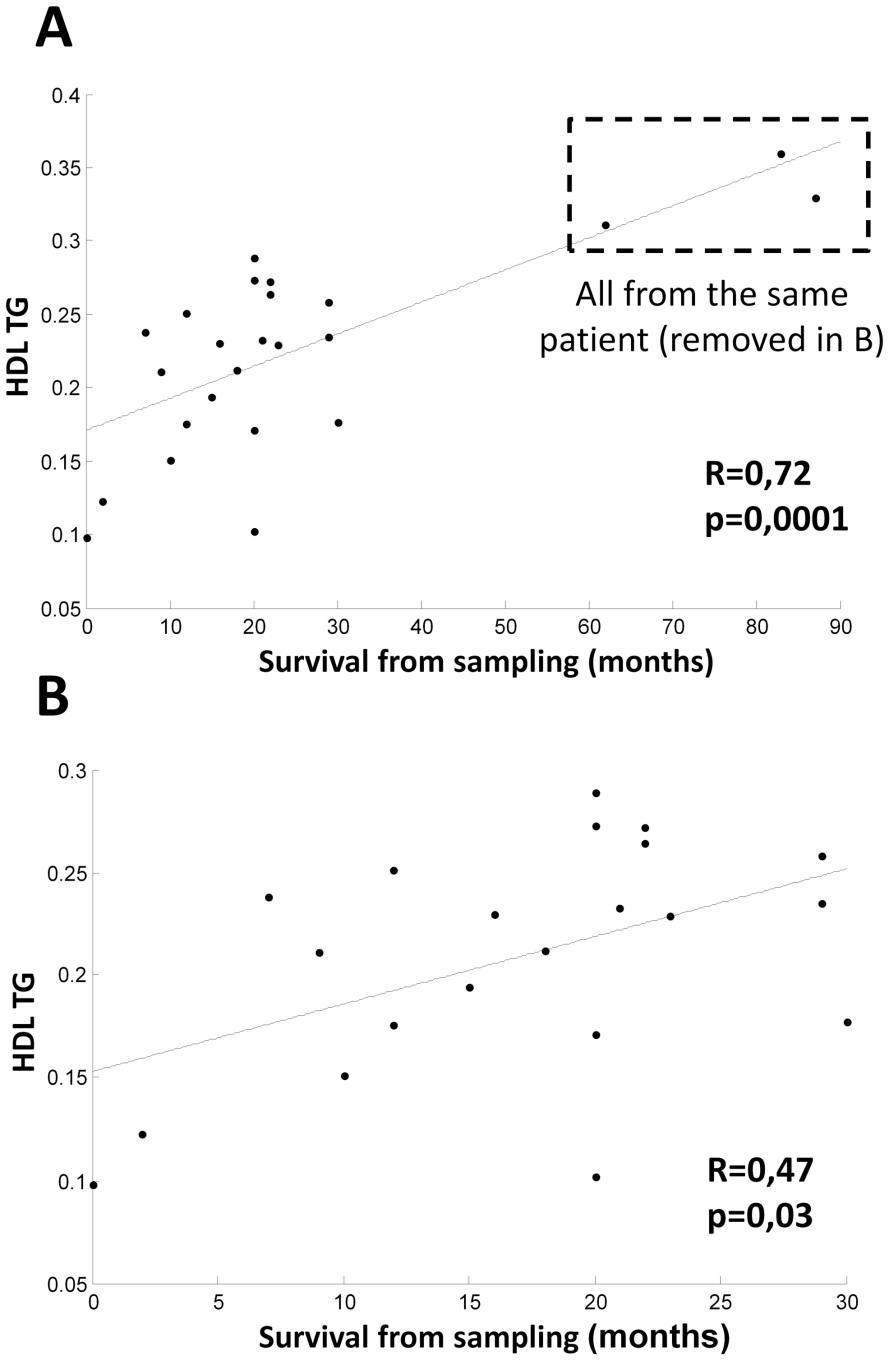
Correlation between HDL triglycerides and time of survival in female patients (A); HDL triglycerides and time of survival in female patients (B) after exclusion of one long-surviving patient (shown in A).

There was a significant correlation between HDL triglyceride levels and time of survival in females (p = 0.0001) (table 3). The correlation between survival and HDL triglycerides (Fig. 2A) was found to be due to one patient with long survival and several samples. However, HDL triglyceride level was still significant (p = 0.03) after exclusion of all the samples from this patient (Fig. 2B). The correlation between HDL triglycerides and survival was, however, not significant if HDL triglyceride level was normalized against age. Lathosterol (p = 0.01) was found to be significantly correlated with survival in the ALS patients, but the correlation was driven by samples from one long-surviving female patient and needs to be further validated in a larger cohort.

The following two parameters were found to be significantly correlated with time of survival in males: LDL cholesterol (p = 0.01), coenzyme Q (p = 0.04), and age (p = 0.04). Also, here the significance was driven by one abnormally long-surviving patient. When this patient was removed, the correlation became non-significant.

**Figure 3:**
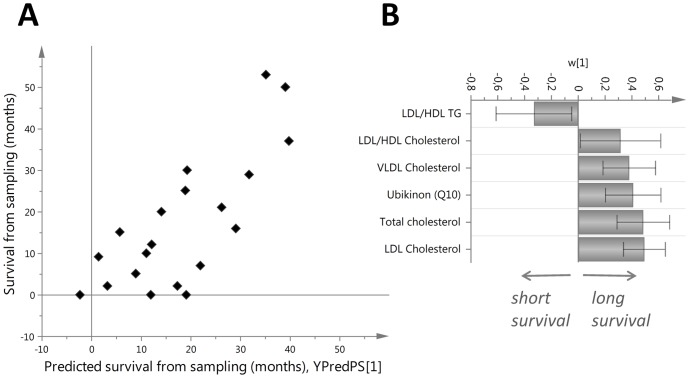
OPLS model predicting survival from the time of sampling for diet-matched ALS cases (p = 0.003). A. Survival predicted by model plotted against time of survival from sampling. B. Long survival from sampling correlated with higher levels of LDL cholesterol, total cholesterol, coenzyme Q, and VLDL cholesterol.

After normalization against diet using the spouse, OPLS modeling of the parameters showed correlation between VLDL cholesterol, LDL cholesterol, total cholesterol, and coenzyme Q with longer survival from the time of sampling (Fig. 3).

**Figure 4:**
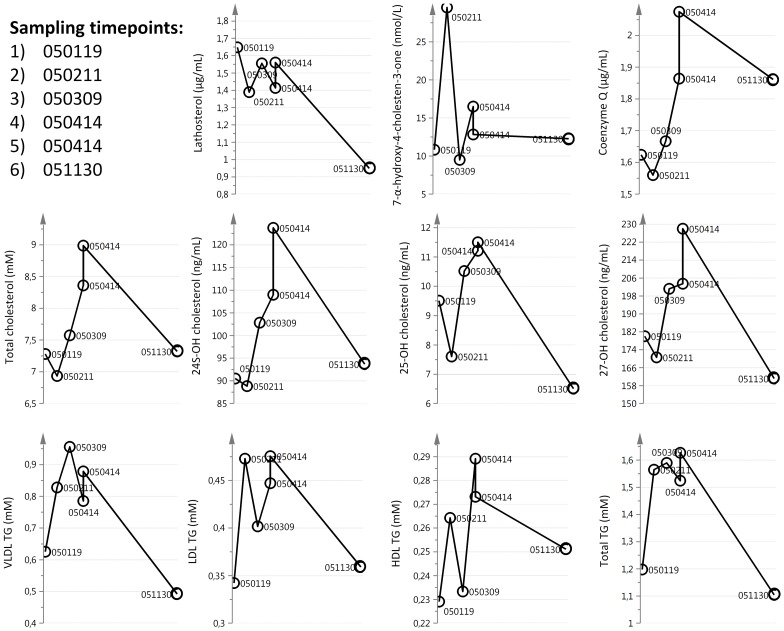
Variability in the measured parameters in one female patient with sporadic pseudo bulbar paresis, the samples were collected over 11 months with one time-point in duplicate.

The results obtained from subjects with several samples collected over time were evaluated for trends. One redundant finding for some but not all patients were decreases in many parameters at the last time point of sampling. High individual variation was seen over time in the patient who was followed over 11 months (Fig. 4).

## Discussion

In accordance with previous investigations [4]–[6], we found a positive correlation between cholesterol levels and survival in ALS. If total cholesterol was normalized against BMI, no significance was found. However, if the corresponding spouse was used for normalization against diet, longer survival was found for increased concentrations of VLDL cholesterol and total cholesterol.

We speculated that cholesterol synthesis rather than cholesterol levels may be important for ALS survival. We did not however find any association between circulating levels of lathosterol, a marker for cholesterol synthesis, and increased survival. Also, there was no correlation between survival and 7α-hydroxy-4-cholesten-3-one, a marker of cholesterol degradation into bile acids [25].

In a previous work, high levels of triglycerides were found to be correlated with increased survival in ALS and high levels of triglycerides were more favorable than high levels of cholesterol [6]. In the present work, a high correlation was found between triglyceride levels in a specific lipoprotein faction, HDL, and survival in females, but not in males (Fig. 2). To some extent the levels of triglycerides are likely to reflect the nutritional status, and according to a previous study, good nutritional status is associated with longer survival time [6]. According to the data presented in tables 1 and 2, the nutritional status of the female ALS patients in the present study can be assumed to have been better than that of the males. The correlation was not statistically significant in the females after normalization of HDL against age or diet. HDL is a cholesterol-rich but triglyceride-poor particle involved in reversed cholesterol transport from peripheral tissues to the liver. It is possible, however, that this fraction is a marker for the nutritional state. In view of the small number of patients, it is difficult to draw firm conclusions from the present finding.

In males, there was a significant correlation between coenzyme Q and survival, which was not significant if adjusted for BMI. However, when the data was normalized against diet using spouses, there was a correlation between higher coenzyme Q levels and longer survival. There was no difference between the controls and ALS patients with respect to coenzyme Q levels, and compensation for the differences in BMI did not result in significant differences.

27-hydroxycholesterol is a metabolite of cholesterol formed in almost all organs and tissues. Since 27-hydroxycholesterol is able to rapidly pass through cell membranes and to be converted into bile acids in the liver, formation of this oxysterol represents an alternative to the classical HDL-mediated reverse cholesterol transport [26]. Surprisingly, the gene coding for the enzyme involved in the conversion of cholesterol into 27-hydroxycholesterol, *CYP27A1*, was recently suggested to be a risk gene for sporadic ALS. If that is the case, one could speculate that altered expression of the enzyme would result in altered plasma levels of its product 27-hydroxycholesterol. That 27-hydroxycholesterol is able to pass the blood-brain barrier and appears to accelerate neurodegeneration by different mechanisms [14,27]–[28] is consistent with 27-hydroxycholesterol being a pathogenic factor. In view of this, we found it to be of interest to study whether the levels of 27-hydroxycholesterol in the circulation are affected in patients with ALS. Contrary to our expectations, however, lower levels were found in male patients than in the corresponding controls. No difference was observed between female ALS patients and controls, and there was no significant correlation between plasma levels of 27-hydroxycholesterol and time of survival. The levels of 27-hydroxycholesterol in the circulation are normally correlated to the levels of cholesterol, and high cholesterol levels in the circulation are associated with high levels of 27-hydroxycholesterol [29]. The levels of 27-hydroxycholesterol are dependent upon the activity of the metabolizing enzyme CYP7B1 in the liver, and it may be speculated that the activity of this enzyme is higher in the male ALS patients than in the controls. The activity of the enzyme CYP7B1 has a sexual dimorphism in experimental animals [30]. Such a dimorphism may be the reason for slightly higher levels of 27-hydroxycholesterol in the circulation of healthy males than in females.

For reasons of comparison, we measured the level of another side-chain oxidized oxysterol, 24S-hydroxycholesterol. This oxysterol is produced by neuronal cells in the brain, and there is a continuous flow of 24S-hydroxycholesterol from the brain into the circulation [14]. In patients with advanced neurodegeneration associated with reduced numbers of neuronal cells, production of this oxysterol is reduced, with lower levels in plasma [31]. ALS affects both the upper motor neurons and the lower motor neurons. Hence, a change in the circulating levels of 24S-hydroxycholesterol would be expected in the plasma of the ALS patients compared to the controls. The levels of 24S-hydroxycholesterol were however only found to be significantly increased in females with ALS but were not significantly different from controls in males and there was no significant correlation with time of survival.

### Conclusions

We conclude that measurements of cholesterol, 24S-, 25- and 27-hydroxycholesterol, and lipoprotein triglyceride profiles in the plasma of individual ALS patients are of limited value from a prognostic point of view. Results for the measured parameters from the same individuals over time clearly showed that the variability over time will pose a problem for clinical use. The results of our study do not support the hypotheses that cholesterol synthesis, 27-hydroxycholesterol or Q10 are pathogenic factors in ALS.

## Supporting Information

File S1 **File includes Figures S1–S6 and Tables S1–S4.**
(DOCX)
