# Supplementary material for: Cholesterol, Oxysterol, Triglyceride, and Coenzyme Q Homeostasis in ALS. Evidence against the Hypothesis That Elevated 27-Hydroxycholesterol Is a Pathogenic Factor
Source: PLoS One. 2014 Nov 21;9(11):e113619. doi: 10.1371/journal.pone.0113619 (PMC4240598; doi:10.1371/journal.pone.0113619)
Supplement: File S1 — File includes Figures S1–S6 and Tables S1–S4. (DOCX) [file pone.0113619.s001.docx]

Supplementary figures and tables

**Fig S1.** BMI and age distribution in the control and ALS groups, subdivided into genetic groups and gender.

**Fig S2.** Coenzyme Q and 7-α -hydroxy-4-cholesten-3-one in the control and ALS groups, subdivided into genetic groups and gender.

**Fig S3.** VLDL-, LDL-, HDL- and total-cholesterol levels in the control and ALS groups, subdivided into genetic groups and gender.

**Fig S4.** 24S-hydroxy cholesterol, 25-hydroxy cholesterol, 27-hydroxy cholesterol and lathosterol in the control and ALS groups, subdivided into genetic groups and gender.

**Fig S5.** VLDL-, LDL-, HDL- and total-triglycerides in the control and ALS groups, subdivided into genetic groups and gender.

**Fig S6.** A multivariate model (OPLS-DA) on male subjects was built on variables VLDL triglycerides, 27-hydroxy cholesterol, VLDL cholesterol, total triglycerides, coenzyme Q, HDL cholesterol and the ratio of LDL/HDL triglycerides. A) Weights [w1] from the OPLS-DA model where grey represent variables with p<0,05. B) Scores from the OPLS-DA model where a difference can be seen between controls (black diamonds) and ALS (grey diamonds). A male patient with a SOD1 (A4V) mutation where one sample was taken 8 years prior ALS diagnosis and the other sample was taken when patient had developed advanced ALS was predicted into the model (marked in red) showing that the sample taken 8 years before diagnosis cluster with ALS. C) The same male patient prior ALS and advanced ALS showed a decreased level of VLDL cholesterol, VLDL triglycerides, total triglycerides D) and also decreased levels of 27-hydroxy cholesterol.

**Table S1.** Patient characteristics.

| **#** | **Diagnosis** | **Geno-**  **typing** | **Sex** | **Age** | **Age at ALS onset (yrs)** | **Survival from sampling (months)** | **Sampling date** | **BMI** | **Drugs and supplements** | **Comments** |
| --- | --- | --- | --- | --- | --- | --- | --- | --- | --- | --- |
| p1 | sALS |  | f | 60 | 46 | 45 | 100602 | 26.4 |  |  |
| p2 | sALS (Vulpian-Bernhardts) |  | f | 75 | 68 | 16 | 070425 | 23.7 |  |  |
| p3 | sALS |  | m | 68 | 66 | 37 | 070620 | 25.9 |  |  |
| p4 | sALS to sPLS |  | f | 52 | 50 | 48 | 100319 | 20.1 |  |  |
| p5 | sALS | C9orf72 | f | 68 | 66 | 15 | 081201 |  |  |  |
| p6 | fALS | C9orf72 | m | 66 | 65 | 5 | 040901 | 27.8 |  |  |
| p7 | sPBP |  | f | 28 | 27 | 9 | 070525 | 19.1 |  |  |
| p8 | fALS | C9orf72 | f | 51 | 50 | 29 | 050429 | 21.8 |  |  |
| p9 | fPBP FD | C9orf72 | f | 56 | 54 | 20 | 050524 | 22.6 |  |  |
| p10 | sALS | C9orf72 | m | 64 | 62 | 21 | 060322 | 24.6 |  |  |
| p11 | fALS | C9orf72 | m | 57 | 56 | 12 | 101117 |  |  |  |
| p12 | fALS | C9orf72 | m | 63 | 58 | 63 | 081210 | 27 |  |  |
| p13 | fALS | C9orf72 | f | 63 | 61 | 22 | 060309 | 24.7 |  |  |
| p14 | sALS | C9orf72 | m | 64 | 63 | 21 | 060411 | 22.6 |  | Autopsy |
| p15 | fALS | C9orf72 | f | 64 | 63 | 7 | 070402 |  |  |  |
| p16 | sALS |  | m | 68 | 67 | 4 | 110221 | 22.2 |  | Autopsy |
| p17 | fALS (PLS-like) | C9orf72 | f | 52 | 51 | 29 | 090909 |  |  |  |
| p18 | fALS |  | f | 73 | 72 | 2 | 060906 |  |  |  |
| p19 | sALS |  | m | 60 | 58 | 90 | 060831 |  |  |  |
| p20 | sPBP FD |  | m | 76 | 75 | 0 | 060316 | 16.3 |  |  |
| p21 | sALS FD |  | m | 80 | 77 | 2 | 050126 | 19.9 |  |  |
| p22 | fALS | SOD1 (G93S) | m | 52 | 51 | alive | 070129 | 30 |  |  |
| **p23** | **sALS** | **SOD1 (D90A)** | **f** | **38** | **38** | **166** | **000519** |  |  |  |
| **p23** | **sALS** | **SOD1 (D90A)** | **f** | **44** | **38** | **101** | **051005** | **25.6** |  |  |
| **p23** | **sALS** | **SOD1 (D90A)** | **f** | **44** | **38** | **98** | **060111** | **23.5** |  |  |
| p24 | sALS |  | m | 65 | 64 | 15 | 050616 | 24 |  |  |
| p25 | sALS | VAPB | m | 44 | 34 | 59 | 090416 |  |  |  |
| p26 | sALS (Vulpian-Bernhardts) |  | f | 61 | 47 | 70 | 080429 | 29.3 |  | Flail arm syndrome. Has a BiPAP |
| p27 | sALS (Vulpian-Bernhardts) |  | m | 63 | 62 | 7 | 101108 | 23.1 |  |  |
| p28 | fALS | SOD1 (D101G) | m | 41 | 39 | 2 | 060125 |  |  |  |
| p29 | sALS | SOD1 (D90A) | m | 68 | 55 | 28 | 060215 | 22.8 |  | Autopsy |
| p30 | sPBP (PLS like) | FUS R521H | m | 44 | 35 | 53 | 091021 | 24.1 |  | Dominating UMN signs but also LMN |
| p31 | sALS |  | m | 38 | 36 | 56 | 090716 | 24.9 |  |  |
| **p32** | **fALS** | **SOD1 (A4V)** | **m** | **54** | **61** | **113** | **960115** |  |  | **Sample drawn 8 years before developing ALS (premorbid)** |
| **p32** | **fALS** | **SOD1 (A4V)** | **m** | **63** | **61** | **1** | **050425** |  |  | **Sample drawn when patient had developed advanced ALS (no PEG or feeding tube)** |
| p33 | sALS |  | f | 54 | 51 | 73 | 080214 |  |  |  |
| p34 | fPBP | FUS (R495X) | f | 17 | 16 | 0 | 060526 | 13.8 |  | Sample drawn when patient had developed advanced ALS (no PEG or feeding tube). Autopsy |
| p35 | sALS |  | m | 70 | 68 | 0 | 070525 | 24.2 |  |  |
| p36 | sPBP FD |  | f | 63 | 61 | 10 | 061206 | 29.4 | Statin |  |
| p37 | fALS |  | f | 63 | 61 | 34 | 110513 | 27 |  |  |
| p38 | sPBP |  | m | 70 | 69 | 25 | 061115 | 24.2 |  |  |
| **p39** | **sALS** |  | **m** | **49** | **48** | **53** | **090818** | **30.9** |  |  |
| **p39** | **sALS** |  | **m** | **49** | **48** | **50** | **091028** |  |  |  |
| p40 | sPBP |  | f | 64 | 63 | 20 | 070426 | 17.5 |  |  |
| p41 | sALS FD |  | m | 65 | 63 | 9 | 061128 | 18.5 |  |  |
| p42 | fPBP | C9orf72 | f | 70 | 69 | 12 | 071129 | 25.3 |  |  |
| p43 | sALS |  | m | 71 | 69 | 0 | 090114 | 24.2 |  |  |
| p44 | sPBP |  | f | 53 | 52 | 30 | 090305 | 23.4 |  |  |
| p45 | sALS |  | m | 37 | 36 | 34 | 081112 | 22.9 |  |  |
| **p46** | **sALS** |  | **f** | **76** | **71** | **87** | **050930** | **22.2** |  |  |
| **p46** | **sALS** |  | **f** | **76** | **71** | **83** | **060125** | **22.2** |  |  |
| **p46** | **sALS** |  | **f** | **78** | **71** | **62** | **071025** | **21.5** |  |  |
| **p47** | **sPBP** |  | **f** | **55** | **54** | **23** | **050119** | **28.2** |  |  |
| **p47** | **sPBP** |  | **f** | **55** | **54** | **22** | **050211** |  |  |  |
| **p47** | **sPBP** |  | **f** | **55** | **54** | **21** | **050309** | **28.2** |  |  |
| **p47** | **sPBP** |  | **f** | **55** | **54** | **20** | **050414** |  |  |  |
| **p47** | **sPBP** |  | **f** | **55** | **54** | **20** | **050414** |  |  |  |
| **p47** | **sPBP** |  | **f** | **56** | **54** | **12** | **051130** | **24** |  |  |
| p48 | sALS |  | m | 75 | 72 | 28 | 090204 | 23 |  |  |
| p49 | fALS | SOD1 (D90A) | f | 67 | 65 | alive | 081217 | 23.9 |  |  |
| p50 | fALS | SOD1 (H46R) | m | 58 | 54 | alive | 070219 |  |  | Slow progression |
| **p51** | **sALS** |  | **m** | **31** | **25** | **61** | **090204** | **28.3** |  |  |
| **p51** | **sALS** |  | **m** | **34** | **25** | **19** | **120820** |  |  |  |
| p52 | sALS (Vulpian-Bernhardts) |  | m | 79 | 78 | 19 | 120823 | 23.9 |  | Slow progression |
| c1 | Control |  | f | 53 |  |  | 060919 | 23.6 |  | No tobacco |
| c2 | Control |  | m | 78 |  |  | 070425 | 26 |  |  |
| c3 | Control |  | f | 65 |  |  | 070620 | 23.9 | Calcium, vitamin D | No tobacco, osteoporosis |
| c4 | Control |  | m | 27 |  |  | 080110 |  |  |  |
| c5* | Control |  | m | 61 |  |  | 080312 |  |  |  |
| c6 | Control |  | f | 63 |  |  | 040331 |  |  |  |
| c7 | Control |  | m | 59 |  |  | 040331 | 29.5 | No drugs, ginger daily | No tobacco |
| c8 | Control |  | f | 67 |  |  | 040901 | 23.5 | No drugs | No tobacco |
| c9 | Control |  | m | 31 |  |  | 070525 |  |  |  |
| c10 | Control |  | f | 64 |  |  | 070525 | 20.6 | No drugs | No tobacco |
| c11 | Control |  | m | 62 |  |  | 061206 |  |  |  |
| c12 | Control |  | f | 65 |  |  | 070507 |  |  |  |
| c13 | Control |  | f | 69 |  |  | 061115 | 23.1 | No drugs | No tobacco |
| c14 | Control |  | f | 63 |  |  | 060322 | 22.8 | No drugs | No tobacco |
| c15 | Control |  | m | 67 |  |  | 070426 | 23.9 | Omega 3 | No tobacco |
| c16 | Control |  | f | 62 |  |  | 081210 | 25.4 | Kandesartancilexetil | No tobacco |
| c17 | Control |  | m | 72 |  |  | 071129 |  |  |  |
| c18 | Control |  | f | 65 |  |  | 090114 | 23.2 |  | No tobacco |
| c19 | Control |  | m | 62 |  |  | 070402 | 30.4 |  | No tobacco |
| c20 | Control |  | m | 55 |  |  | 090305 | 27.4 |  | Snus |
| c21 | Control |  | m | 55 |  |  | 090909 |  |  |  |
| c22 | Control |  | m | 76 |  |  | 060906? | 28.3 |  | No tobacco, diabetes |
| c23 | Control |  | f | 59 |  |  | 060831 | 24.5 | Simvastatin,metoprolol,kandesartancilexeti | No tobacco |
| c24 | Control |  | f | 72 |  |  | 060316 | 26.2 | Simvastatin, propranolol | No tobacco |
| c25 | Control |  | f | 64 |  |  | 050128 | 16.8 | No drugs | Smoker |
| c26 | Control |  | m | 59 |  |  | 050524 |  |  |  |
| c27 | Control |  | f | 66 |  |  | 050616 |  |  |  |
| c28 | Control |  | f | 72 |  |  | 120823 |  |  |  |
| c29 | Control |  | f | 74 |  |  | 091014 | 29.3 | Bendroflumetazid | No tobacco |
| c30 | Control |  | f | 53 |  |  | 091021 | 20.1 | No drugs | No tobacco |
| c31 | Control |  | f | 50 |  |  | 091028 |  |  |  |
| c32 | Control |  | m | 64 |  |  | 100312 | 26.6 | No drugs | Snus |
| c33 | Control |  | m | 60 |  |  | 100909 | 32.7 | Atenolol | Smoker |
| c34 | Control |  | m | 57 |  |  | 101108 | 24.7 | No drugs | No tobacco |
| c35 | Control |  | m | 68 |  |  | 110513 | 27.4 | Levotyroxin, acetylsalicylic acid, bendroflumetiazid, losartan | No tobacco |
| c36* | Control |  | m | 62 |  |  | 110527 | 27.1 | Simvastatin, acetylsalicylic acid, atenolol | No tobacco |
| c37 | Control |  | m | 52 |  |  | 111123 | 26.9 | Loperamid | No tobacco |
| c38* | Control |  | m | 67 |  |  | 120515 | 22.5 | No drugs | No tobacco |
| c39* | Control |  | m | 58 |  |  | 120821 | 29.7 | No drugs | No tobacco |
| c40* | Control |  | f | 67 |  |  | 120620 |  |  |  |
| *** | *not a spouse* |  |  |  |  |  |  |  |  |  |
| **Bold** | *same individual over time* |  |  |  |  |  |  |  |  |  |

**Table S2.** Analytical data and spouse information.

| ID | Spouse | 1 | 2 | 3 | 4 | 5 | 6 | 7 | 8 | 9 | 10 | 11 | 12 |
| --- | --- | --- | --- | --- | --- | --- | --- | --- | --- | --- | --- | --- | --- |
| p1 | 0 | 0.693 | 0.241 | 0.199 | 0.963 | 2.409 | 2.223 | 1.01 | 12.9 | 46.1 | 5.7 | 92 | 1.7 |
| p2 | 12 | 1.282 | 0.278 | 0.23 | 1.308 | 3.232 | 1.656 | 1.65 | 54 | 76.9 | 4 | 139 | 1.3 |
| p3 | 16 | 1.023 | 0.275 | 0.223 | 0.923 | 5.138 | 1.487 | 1.45 | 116 | 81.7 | 11 | 234 | 2.5 |
| p4 | 0 | 0.212 | 0.346 | 0.207 | 0.291 | 2.758 | 1.812 | 0.91 | 28.3 | 56.8 | 5 | 143 | 1.4 |
| p5 | 0 | 0.848 | 0.287 | 0.194 | 0.997 | 3.667 | 1.341 | 1.43 | 81.8 | 91 | 16 | 247 | 2 |
| p6 | 1 | 0.944 | 0.285 | 0.214 | 0.756 | 2.852 | 1.012 | 1.47 | 103 | 40.2 | 6.7 | 122 | 1.8 |
| p7 | 14 | 0.175 | 0.421 | 0.211 | 0.145 | 3.087 | 2.523 | 0.69 | 42.2 | 65.4 | 1.3 | 90 | 1.3 |
| p8 | 0 | 0.444 | 0.237 | 0.258 | 0.593 | 3.045 | 2.291 | 1.49 | 42.5 | 55.2 | 7.1 | 119 | 1.3 |
| p9 | 3 | 0.595 | 0.258 | 0.171 | 0.742 | 3.8 | 1.768 | 0.48 | 8.6 | 57.3 | 4.7 | 135 | 1.3 |
| p10 | 6 | 0.411 | 0.269 | 0.121 | 0.533 | 4.292 | 1.598 | 0.88 | 40.9 | 59.5 | 9.4 | 124 | 1.6 |
| p11 | 0 | 0.479 | 0.289 | 0.204 | 1.224 | 4.006 | 1.354 | 1.18 | 64.1 | 62.8 | 6.7 | 153 | 1.8 |
| p12 | 18 | 0.755 | 0.312 | 0.296 | 0.881 | 3.221 | 1.956 | 1.2 | 92.2 | 60.3 | 9.3 | 210 | 2 |
| p13 | 0 | 0.721 | 0.388 | 0.272 | 0.693 | 3.833 | 1.67 | 1.21 | 54.1 | 64.8 | 9 | 137 | 1.4 |
| p14 | 0 | 0.355 | 0.328 | 0.173 | 0.521 | 3.45 | 1.203 | 0.78 | 42.1 | 52.6 | 7 | 114 | 1.1 |
| p15 | 11 | 0.887 | 0.327 | 0.238 | 0.961 | 3.42 | 1.474 | 1.09 | 186 | 63.8 | 7.2 | 140 | 1.5 |
| p16 | 0 | 0.266 | 0.387 | 0.132 | 0.193 | 1.791 | 0.828 | 1.83 | 22.1 | 36.3 | 4.7 | 114 | 1.3 |
| p17 | 21 | 1.298 | 0.321 | 0.235 | 2.412 | 5.694 | 2.42 | 1.03 | 80.1 | 98.7 | 12 | 167 | 2 |
| p18 | 8 | 0.303 | 0.585 | 0.123 | 0.459 | 4.45 | 1.143 | 0.68 | 41.1 | 82.7 | 12 | 127 | 1.5 |
| p19 | 7 | 0.382 | 0.34 | 0.128 | 0.533 | 4.498 | 1.684 | 0.83 | 12.2 | 74.7 | 10 | 247 | 2.7 |
| p20 | 5 | 0.249 | 0.446 | 0.297 | 0.283 | 2.609 | 1.677 | 0.37 | 30.4 | 82.1 | 12 | 231 | 1.2 |
| p21 | 2 | 0.219 | 0.225 | 0.168 | 0.49 | 3.039 | 1.758 | 0.49 | 45.1 | 55.9 | 9.7 | 157 | 1.2 |
| p22 | 0 | 0.816 | 0.243 | 0.209 | 0.67 | 2.759 | 1.166 | 0.79 | 33.6 | 43.1 | 3.2 | 125 | 2.1 |
| p23 | 0 | 0.246 | 0.165 | 0.169 | 0.331 | 1.94 | 2.383 | 0.57 | 26.4 | 45.7 | 2.5 | 106 | 0.8 |
| p23 | 0 | 0.149 | 0.24 | 0.162 | 0.215 | 2.065 | 1.997 | 0.62 | 13.6 | 41.7 | 6.8 | 82 | 1 |
| p23 | 0 | 0.203 | 0.173 | 0.141 | 0.306 | 2.462 | 2.134 | 0.6 | 25.1 | 47 | 7.2 | 90 | 1.3 |
| p24 | 4 | 0.483 | 0.247 | 0.177 | 0.758 | 4.027 | 1.777 | 1.11 | 18.7 | 49.5 | 6.6 | 115 | 1.2 |
| p25 | 0 | 0.215 | 0.261 | 0.193 | 1.021 | 3.641 | 1.754 | 0.71 | 22.1 | 87.9 | 5.3 | 114 | 1.3 |
| p26 | 0 | 0.337 | 0.371 | 0.174 | 0.582 | 3.646 | 3.496 | 1.25 | 48.7 | 91 | 7.5 | 133 | 1.8 |
| p27 | 0 | 1.488 | 0.47 | 0.238 | 2.053 | 4.241 | 1.143 | 0.99 | 80 | 73.2 | 11 | 185 | 2.1 |
| p28 | 0 | 0.551 | 0.506 | 0.143 | 0.706 | 3.223 | 1.062 | 1.85 | 18.6 | 54.8 | 12 | 126 | 1.4 |
| p29 | 0 | 0.328 | 0.142 | 0.128 | 0.522 | 2.424 | 1.743 | 0.8 | 27.9 | 79.8 | 3.7 | 107 | 1.2 |
| p30 | 0 | 0.751 | 0.189 | 0.224 | 0.745 | 3.182 | 1.161 | 1.13 | 24.8 | 66.1 | 7.4 | 128 | 1.7 |
| p31 | 0 | 0.271 | 0.192 | 0.169 | 0.571 | 2.433 | 2.067 | 2.44 | 31.8 | 58.1 | 7.1 | 112 | 1.3 |
| p32 | 0 | 0.611 | 0.344 | 0.235 | 1.141 | 5.61 | 1.998 | 1.69 | 37.7 | 93.4 | 16 | 260 | 1.6 |
| p32 | 0 | 0.238 | 0.293 | 0.165 | 0.643 | 3.679 | 2.493 | 0.82 | 97.2 | 89.9 | 18 | 241 | 1.6 |
| p33 | 0 | 0.79 | 0.207 | 0.274 | 0.872 | 3.097 | 1.913 | 1.09 | 48.2 | 54.3 | 4.6 | 153 | 1.7 |
| p34 | 0 | 0.08 | 0.236 | 0.098 | 0.148 | 2.221 | 1.446 | 0.2 | 24.2 | 91.8 | 4.2 | 135 | 1 |
| p35 | 15 | 0.38 | 0.385 | 0.144 | 0.324 | 2.125 | 1.305 | 0.37 | 6.4 | 47.3 | 8.5 | 67 | 1 |
| p36 | 10 | 0.602 | 0.259 | 0.151 | 0.589 | 2.524 | 1.972 | 1.77 | 36.3 | 58.2 | 6 | 112 | 1.5 |
| p37 | 0 | 1.086 | 0.299 | 0.212 | 1.681 | 4.283 | 1.501 | 1.62 |  | 77.6 | 8.5 | 189 | 1.6 |
| p38 | 9 | 0.488 | 0.587 | 0.432 | 0.451 | 3.463 | 2.48 | 0.54 | 6.9 | 54.9 | 9.6 | 176 | 1.9 |
| p39 | 22 | 0.638 | 0.258 | 0.16 | 0.995 | 3.791 | 1.073 | 1.64 | 31 | 72.8 | 6.2 | 139 | 2 |
| p39 | 22 | 0.655 | 0.255 | 0.127 | 0.648 | 4.329 | 1.384 | 1.71 | 41 | 60 | 5.8 | 124 | 1.6 |
| p40 | 0 | 0.154 | 0.182 | 0.102 | 0.757 | 5.391 | 3.527 | 0.77 | 13.3 | 82.4 | 11 | 196 | 1 |
| p41 | 0 | 0.501 | 0.241 | 0.153 | 0.661 | 4.287 | 1.888 | 0.76 | 45.9 | 54.4 | 7.9 | 181 | 1.8 |
| p42 | 17 | 0.439 | 0.29 | 0.176 | 0.451 | 2.898 | 2.137 | 3.03 | 107 | 65.4 | 6.1 | 136 | 1.9 |
| p43 | 19 | 0.633 | 0.266 | 0.218 | 0.807 | 3.201 | 1.842 | 1.94 | 41.7 | 60.3 | 8.3 | 165 | 1.3 |
| p44 | 20 | 0.32 | 0.234 | 0.177 | 0.833 | 3.749 | 2.807 | 1.07 | 5.3 | 55.7 | 3.8 | 128 | 1.2 |
| p45 | 0 | 0.225 | 0.206 | 0.142 | 0.322 | 2.164 | 2.11 | 0.66 | 7.5 | 46.1 | 3.3 | 167 | 1.5 |
| p46 | 0 | 0.733 | 0.353 | 0.329 | 0.753 | 2.918 | 2.611 | 2.78 | 82.2 | 96.7 | 7.8 | 197 | 1.6 |
| p46 | 0 | 0.677 | 0.347 | 0.36 | 1.036 | 2.588 | 1.865 | 1.62 | 45.4 | 97.1 | 4.2 | 168 | 1.6 |
| p46 | 0 | 1.081 | 0.288 | 0.311 | 0.854 | 2.675 | 2.043 | 1.86 | 52.3 | 91.8 | 7.9 | 167 | 1.5 |
| p47 | 0 | 0.625 | 0.342 | 0.229 | 0.939 | 4.351 | 1.979 | 1.65 | 10.8 | 90.4 | 9.5 | 180 | 1.6 |
| p47 | 0 | 0.826 | 0.472 | 0.264 | 0.697 | 4.442 | 1.791 | 1.39 | 29.5 | 88.7 | 7.6 | 171 | 1.6 |
| p47 | 0 | 0.954 | 0.401 | 0.233 | 1.385 | 4.776 | 1.401 | 1.56 | 9.4 | 102.7 | 11 | 201 | 1.7 |
| p47 | 0 | 0.785 | 0.447 | 0.289 | 1.153 | 5.018 | 2.181 | 1.41 | 16.4 | 108.8 | 11 | 203 | 1.9 |
| p47 | 0 | 0.878 | 0.475 | 0.273 | 1.262 | 5.426 | 2.286 | 1.56 | 12.8 | 123.6 | 12 | 228 | 2.1 |
| p47 | 0 | 0.492 | 0.359 | 0.251 | 0.804 | 4.169 | 2.335 | 0.95 | 12.2 | 93.6 | 6.5 | 161 | 1.9 |
| p48 | 0 | 0.613 | 0.193 | 0.176 | 0.976 | 3.573 | 1.515 | 0.92 | 19.7 | 65.6 | 7.4 | 146 | 1.6 |
| p49 | 0 | 0.923 | 0.275 | 0.277 | 0.604 | 2.744 | 2.146 | 0.41 | 40.9 | 69.5 | 7 | 131 | 1.9 |
| p50 | 0 | 2.01 | 0.334 | 0.262 | 1.245 | 3.328 | 1.07 | 1.77 | 234 | 88.3 | 11 | 271 | 1.6 |
| p51 | 0 | 0.881 | 0.181 | 0.189 | 1.068 | 2.92 | 1.761 | 1.89 | 31.3 | 71.7 | 6.6 | 144 | 1.4 |
| p51 | 0 | 0.746 | 0.31 | 0.326 | 0.837 | 2.389 | 1.202 | 3.25 | 58.1 | 98 | 7.8 | 185 | 1.5 |
| p52 | 0 | 0.357 | 0.257 | 0.191 | 0.49 | 2.318 | 2.263 | 0.84 | 42.8 | 65.8 | 10 | 170 | 1.5 |
| c1 | 0 | 0.091 | 0.177 | 0.109 | 0.194 | 2.314 | 3.363 | 0.98 | 16 | 46.3 | 6.5 | 121 | 1.1 |
| c2 | 12 | 0.954 | 0.163 | 0.17 | 1.192 | 1.349 | 1.509 | 1.09 | 13.3 | 51.4 | 4.3 | 197 | 1.7 |
| c3 | 16 | 0.522 | 0.299 | 0.216 | 0.765 | 2.888 | 2.216 | 1.18 | 21.5 | 59.3 | 3.1 | 115 | 1.5 |
| c4 | 0 | 0.266 | 0.183 | 0.108 | 0.311 | 2.175 | 1.224 | 0.76 | 25 | 64.1 | 4.2 | 112 | 0.9 |
| c5 | 0 | 1.032 | 0.231 | 0.185 | 0.671 | 2.956 | 1.243 | 1.58 | 23.4 | 49.1 | 8.7 | 184 | 1.8 |
| c6 | 0 | 1.692 | 0.249 | 0.223 | 1.235 | 2.332 | 0.906 | 2.93 | 95.6 | 55.5 | 7.1 | 154 | 1.6 |
| c7 | 0 | 0.769 | 0.258 | 0.21 | 1.151 | 4.177 | 1.542 | 1.19 | 67.3 | 63.1 | 7.7 | 247 | 1.7 |
| c8 | 1 | 0.393 | 0.356 | 0.196 | 1 | 4.92 | 1.897 | 0.73 | 53.5 | 68.9 | 5.8 | 164 | 1.1 |
| c9 | 14 | 1.031 | 0.231 | 0.195 | 1.252 | 3.499 | 1.343 | 2.37 | 104 | 70.1 | 6.9 | 163 | 1.7 |
| c10 | 15 | 0.889 | 0.305 | 0.32 | 0.619 | 3.706 | 2.498 | 1.54 | 90.6 | 70.6 | 8.3 | 161 | 1.4 |
| c11 | 10 | 0.803 | 0.236 | 0.229 | 0.79 | 3.04 | 1.329 | 1.94 | 58.7 | 67.1 | 3.7 | 156 | 1.5 |
| c12 | 0 | 1.012 | 0.232 | 0.184 | 0.82 | 2.471 | 1.249 | 0.29 | 34.5 | 47.5 | 7.5 | 130 | 1.5 |
| c13 | 9 | 0.786 | 0.242 | 0.189 | 1.161 | 2.913 | 1.343 | 0.56 | 11.4 | 67 | 5.1 | 135 | 1.3 |
| c14 | 6 | 0.213 | 0.234 | 0.149 | 0.379 | 2.984 | 3.908 | 0.6 | 65.9 | 62.8 | 4.1 | 116 | 1.1 |
| c15 | 0 | 0.582 | 0.317 | 0.19 | 0.813 | 3.576 | 1.132 | 2.54 | 44.2 | 54.5 | 5.2 | 195 | 1.8 |
| c16 | 18 | 0.568 | 0.273 | 0.274 | 1.304 | 3.468 | 2.862 | 0.95 | 14.9 |  |  |  | 1.5 |
| c17 | 17 | 0.579 | 0.263 | 0.197 | 0.686 | 3.473 | 2.023 | 1.01 | 16.9 | 53.6 | 6.4 | 174 | 1.8 |
| c18 | 19 | 0.195 | 0.24 | 0.176 | 0.44 | 2.977 | 2.262 | 1.03 | 13.8 | 53.4 | 6.8 | 148 | 2.1 |
| c19 | 11 | 0.87 | 0.155 | 0.189 | 0.967 | 2.442 | 1.218 | 0.84 | 37.5 | 40.1 | 4.5 | 129 | 1.7 |
| c20 | 20 | 1.535 | 0.287 | 0.33 | 1.31 | 2.418 | 0.959 | 1.33 | 106 | 78.6 | 8.3 | 213 | 1.6 |
| c21 | 21 | 0.773 | 0.379 | 0.238 | 1.856 | 4.088 | 1.505 | 2.55 | 37.7 | 72.8 | 7.1 | 233 | 2.4 |
| c22 | 8 | 1.164 | 0.216 | 0.155 | 0.568 | 2.696 | 1.3 | 0.58 | 89.9 | 34.6 | 7.8 | 142 | 2 |
| c23 | 7 | 0.829 | 0.219 | 0.16 | 0.833 | 2.979 | 1.77 | 0.87 | 24.8 | 54.8 | 4.1 | 171 | 1.4 |
| c24 | 5 | 0.437 | 0.228 | 0.218 | 0.772 | 2.712 | 1.785 | 0.44 | 7.1 | 91.8 | 5.3 | 138 | 1.1 |
| c25 | 2 | 0.689 | 0.343 | 0.241 | 0.94 | 4.16 | 1.885 | 0.8 | 12.3 | 77.6 | 9.6 | 209 | 2 |
| c26 | 3 | 0.833 | 0.229 | 0.159 | 0.734 | 3.583 | 1.323 | 0.86 | 28.9 | 55.1 | 5.9 | 252 | 2.2 |
| c27 | 4 | 1.194 | 0.297 | 0.186 | 1.15 | 4.48 | 1.289 | 1.62 | 36.4 | 58.1 | 7.7 | 125 | 2.3 |
| c28 | 0 | 0.58 | 0.262 | 0.168 | 0.519 | 2.314 | 1.353 | 0.66 | 25.6 | 36.5 | 3.9 | 83 | 1.7 |
| c29 | 0 | 0.745 | 0.218 | 0.192 | 0.792 | 2.359 | 1.942 | 1.05 | 75.1 | 58.9 | 6.4 | 109 | 1.5 |
| c30 | 0 | 0.377 | 0.218 | 0.182 | 0.59 | 3.699 | 3.012 | 1.53 | 61.5 | 70 | 7.3 | 141 | 1.5 |
| c31 | 22 | 0.215 | 0.247 | 0.122 | 0.333 | 2.486 | 1.775 | 0.89 | 19.7 | 49.3 | 4.7 | 127 | 1.3 |
| c32 | 0 | 0.538 | 0.274 | 0.245 | 0.69 | 3.535 | 1.676 | 0.98 | 37.3 | 61.6 | 8.7 | 207 | 2.1 |
| c33 | 0 | 1.032 | 0.257 | 0.188 | 1.044 | 3.065 | 0.935 | 1.18 | 28.1 | 65 | 8.3 | 182 | 1.6 |
| c34 | 0 | 0.845 | 0.22 | 0.225 | 1.097 | 3.653 | 1.929 | 0.58 | 95.8 | 59.8 | 6 | 138 | 1.6 |
| c35 | 0 | 0.861 | 0.246 | 0.202 | 0.936 | 2.707 | 1.242 | 1.73 | 29.8 | 63.5 | 7.2 | 222 | 1.7 |
| c36 | 0 | 1.406 | 0.521 | 0.346 | 1.646 | 3.097 | 1.187 | 0.56 | 20.1 | 87.1 | 12 | 271 | 1.7 |
| c37 | 0 | 1.501 | 0.271 | 0.243 | 1.527 | 3.681 | 1.319 | 2.93 | 452 | 79 | 13 | 277 | 1.9 |
| c38 | 0 | 0.471 | 0.207 | 0.159 | 0.904 | 4.044 | 2 | 1.14 | 37.5 | 64.4 | 11 | 180 | 1.6 |
| c39 | 0 | 0.744 | 0.531 | 0.402 | 1.119 | 3.452 | 1.441 | 3.26 | 79.3 | 53.1 | 11 | 283 | 2.3 |
| c40 | 0 | 0.209 | 0.302 | 0.138 | 0.455 | 3.771 | 2.93 | 0.69 | 34.7 | 86.2 | 12 | 154 | 1.4 |

Spouse: 0=spouses to ALS from other household; matching numbers= spouses from the same household.
Var 1= Very low density lipoprotein triglyceride fraction (mM)
Var 2= Low density lipoprotein triglyceride fraction (mM)
Var 3= High density lipoprotein triglyceride fraction (mM)
Var 4= Very low density lipoprotein cholesterol fraction (mM)
Var 5= Low density lipoprotein cholesterol fraction (mM)
Var 6= High density lipoprotein cholesterol fraction (mM)
Var 7= Lathosterol (µg/mL)
Var 8= 7-alfa-hydroxy-4-cholesten-3-one (nmol/L)
Var 9= 24S-hydroxy cholesterol (ng/mL)
Var 10= 25-hydroxy cholesterol (ng/mL)
Var 11= 27-hydroxy cholesterol (ng/mL)
Var 12= Ubikinon (Q10) (µg/mL)

**Table S3.** Mann-Whitney U test p-values before and after age normalization in females (adjusting for differences between females in age, fold 0.9 ALS/controls) and BMI normalization in the males (adjusting for differences between males in BMI, fold 0.9 ALS/controls).

| **Name of analyte** | **p(females)**  Mann-Whitney U test | | **p(males)**  Mann-Whitney U test | |
| --- | --- | --- | --- | --- |
|  |  | *Age adjusted* |  | *BMI adjusted* |
| Total cholesterol | 0.37 | 0.09 | 0.79 | 0.26 |
| VLDL cholesterol | 0.94 | 0.80 | **0.01** (**↓** in ALS**)** | **0.00** (**↓**in ALS**)** |
| LDL cholesterol | 0.23 | **0.04** (**↑**in ALS**)** | 0.76 | 0.21 |
| HDL cholesterol | 0.69 | 0.25 | 0.18 | **0.03** (**↑**in ALS**)** |
| Total triglycerides | 0.70 | 0.09 | **0.01** (**↓** in ALS**)** | **0.05** (**↓**in ALS**)** |
| VLDL triglycerides | 0.95 | 0.58 | **0.00** (**↓** in ALS**)** | **0.00** (**↓**in ALS**)** |
| LDL triglycerides | 0.11 | **0.01** (**↑**in ALS**)** | 0.20 | 0.22 |
| HDL triglycerides | 0.25 | **0.01** (**↑**in ALS**)** | 0.22 | 0.70 |
| Lathosterol | 0.23 | **0.05** (**↑**in ALS**)** | 0.18 | 0.72 |
| 7 alfa-hydroxy-4-cholesten-3-one | 0.43 | 0.20 | 0.30 | 0.33 |
| 24S-hydroxy cholesterol | 0.12 | **0.00** (**↑**in ALS**)** | 0.67 | 0.31 |
| 25-hydroxy cholesterol | 0.66 | 0.18 | 0.39 | 0.57 |
| 27-hydroxy cholesterol | 0.77 | 0.08 | **0.01** **(↓** in ALS**)** | **0.03** (**↓**in ALS**)** |
| Coenzyme Q | 0.63 | 0.07 | 0.07 | 0.87 |

**Table S4.** Significant correlations between age and the measured parameters and significant correlations between BMI and the measured parameters calculated for female and male controls and ALS.

| **Significant correlations (p<0,05)** | **R** | **Disease** | **Gender** |
| --- | --- | --- | --- |
| Age x VLDL triglycerides | 0.43 | ALS | females |
| Age x Total triglycerides | 0.48 | ALS | females |
| Age x Lathosterol | 0.45 | ALS | females |
| Age x Total triglycerides | 0.48 | ALS | females |
| Age x Coenzyme Q | 0.44 | ALS | females |
| Age x 7 alfa-hydroxy-4-cholesten-3-one | 0.44 | ALS | females |
| Age x 25-hydroxycholesterol | 0.46 | ALS | females |
| BMI x VLDL triglycerides | 0.46 | ALS | females |
| BMI x Lathosterol | 0.46 | ALS | females |
| BMI x Coenzyme Q | 0.58 | ALS | females |
| Age x BMI | -0.38 | ALS | males |
| Age x Lathosterol | -0.37 | ALS | males |
| BMI x Age | -0.38 | ALS | males |
| BMI x VLDL triglycerides | 0.55 | ALS | males |
| BMI x Total triglycerides | 0.48 | ALS | males |
| BMI x Lathosterol | 0.44 | ALS | males |
| BMI x Ubiquinone (Q10) | 0.43 | ALS | males |
| BMI x 27-hydroxycholesterol | -0.55 | Controls | females |
